# Nonvolatile, Reconfigurable and Narrowband Mid-Infrared Filter Based on Surface Lattice Resonance in Phase-Change Ge_2_Sb_2_Te_5_

**DOI:** 10.3390/nano10122530

**Published:** 2020-12-16

**Authors:** Xingzhe Shi, Changshui Chen, Songhao Liu, Guangyuan Li

**Affiliations:** 1 Guangdong Provincial Key Laboratory of Nanophotonic Functional Materials and Devices, School of Information Optoelectronic Science and Engineering, South China Normal University, Guangzhou 510006, China; xz.shi@siat.ac.cn (X.S.); cschen@aiofm.ac.cn (C.C.); liush@scnu.edu.cn (S.L.); 2 CAS Key Laboratory of Human-Machine Intelligence-Synergy Systems, Shenzhen Institutes of Advanced Technology, Chinese Academy of Sciences, Shenzhen 518055, China; 3 Guangdong-Hong Kong-Macao Joint Laboratory of Human-Machine Intelligence-Synergy Systems, Chinese Academy of Sciences, Shenzhen Institutes of Advanced Technology, Shenzhen 518055, China; 4 Shenzhen College of Advanced Technology, University of Chinese Academy of Sciences, Shenzhen 518055, China

**Keywords:** plasmonics, surface lattice resonance, phase-change materials, min-infrared filter, reconfigurable tuning

## Abstract

We propose a nonvolatile, reconfigurable, and narrowband mid-infrared bandpass filter based on surface lattice resonance in phase-change material Ge${}_{2}$Sb${}_{2}$Te${}_{5}$. The proposed filter is composed of a two-dimensional gold nanorod array embedded in a thick Ge${}_{2}$Sb${}_{2}$Te${}_{5}$ film. Results show that when Ge${}_{2}$Sb${}_{2}$Te${}_{5}$ transits from the amorphous state to the crystalline state, the narrowband reflection spectrum of the proposed filter is tuned from 3.197 μm to 4.795 μm, covering the majority of the mid-infrared regime, the peak reflectance decreases from 72.6% to 25.8%, and the corresponding quality factor decreases from 19.6 to 10.3. We show that the spectral tuning range can be adjusted by varying the incidence angle or the lattice period. By properly designing the gold nanorod sizes, we also show that the quality factor can be greatly increased to 70 at the cost of relatively smaller peak reflection efficiencies, and that the peak reflection efficiency can be further increased to 80% at the cost of relatively smaller quality factors. We expect that this work will advance the engineering of Ge${}_{2}$Sb${}_{2}$Te${}_{5}$-based nonvalatile tunable surface lattice resonances and will promote their applications especially in reconfigurable narrowband filters.

## 1. Introduction

Dynamically tunable narrowband mid-infrared (3 μm to 5 μm) filters are key devices in a diverse range of applications, including chemical spectroscopy, thermography, multispectral/hyperspectral imaging [1,2,3]. It has been accepted that conventional tunable narrowband mid-infrared filters realized by using diffraction gratings, motorized filter wheels, acousto-optic interactions [4,5], or Fabry-Pérot interferometers based on micro-electro-mechanical systems [6] or liquid-crystals [7] suffer from different limitations, as summarized by Julian et al. [8]. For example, diffraction gratings and motorized filter wheels have moving parts and slow tuning speed, acousto-optic tunable filters are complex to manufacture and integrate, and Fabry-Pérot interferometer-based filters have limited spectral tunability.

Recently, chalcogenide phase-change materials, especially germanium–antimony–telluride (Ge${}_{x}$Sb${}_{y}$Te${}_{z}$), have attracted increasing attention because of their appealing merits such as nonvolatile, rapid and reversible switching between the amorphous and the crystalline states by electrical or short optical pulses, tremendous differences in optical and electronic properties between these two states, and high chemical and long-term stability [9,10,11,12]. Based on Ge${}_{x}$Sb${}_{y}$Te${}_{z}$ as a nonvolatile reconfigurable platform, various tunable mid-infrared filters have been proposed or demonstrated. Cao et al. [13,14], Tittl et al. [15], and Tian et al. [16] respectively proposed or demonstrated tunable mid-infrared filters based on perfect absorbers that are composed of one- or two-dimensional metal metasurface on top of a metallic mirror sandwiched by a Ge${}_{x}$Sb${}_{y}$Te${}_{z}$ film. However, only bandstop (or notch) filters in reflection mode can be realized based on perfect absorbers, and the spectral tunability is limited (only ∼300 nm in [13], ∼500 nm in [15], 1300 nm in [14], or 1464 nm in [16]). Ding et al. [17] also proposed tunable bandstop mid-infrared filters based on Ge${}_{2}$Sb${}_{2}$Te${}_{5}$ metasurface composed of nanorods. In order to achieve bandpass filters, Zhou et al. [18] and Williams et al. [19] respectively proposed or demonstrated tunable mid-infrared bandpass filters by embedding a Ge${}_{2}$Sb${}_{2}$Te${}_{5}$ film in a Fabry-Pérot cavity. Although the quality factor, defined as the ratio of the linewidth to the central wavelength, can reach 70–90, the spectral tunability is very limited (only ∼420 nm in [18] or ∼300 nm in [19]). Rudé et al. [20] combined Ge${}_{2}$Sb${}_{2}$Te${}_{5}$ thin films with extraordinary optical transmission through periodic arrays of subwavelength nanoholes drilled in a thick gold film, and demonstrated that the resonant wavelength can shift by 385 nm. Later Trimby et al. [21] discussed approaches to modify the filter performance, including the quality factor, the spectral range, and the peak transmission. Quite recently, Julian et al. [8] demonstrated reversible mid-infrared filters with high transmittance (∼70%) and narrowband performance (Q∼45). However, the spectral tunability (500 nm, from 2.91 μm to 3.41 μm) is still too small to cover the mid-infrared regime.

Recently, plasmonic surface lattice resonances (SLRs), which are collective Fano resonances formed by the diffraction coupling of localized surface plasmon resonance [22,23,24], have been of particular interest in ultra-narrowband absorbing/filtering applications [25]. In 2013, Chen et al. [26] demonstrated nonvolatile tuning of SLRs over a range of ∼500 nm in the near-infrared regime (1.89 μm to 2.27 μm) by incorporating a thin Ge${}_{2}$Sb${}_{2}$Te${}_{5}$ film between a gold nanodisk array and a quartz substrate. However, the obtained transmission spectra profiles are not suitable for narrowband filtering applications. Michel et al. [27] demonstrated tunable filters by combining thin Ge${}_{3}$Sb${}_{2}$Te${}_{6}$ films with nanoantennas that support SLRs, and achieved resonant wavelength shift up to 668 nm (from 3.926 μm to 4.594 μm) when the Ge${}_{3}$Sb${}_{2}$Te${}_{6}$ phase transits from the amorphous state to the crystalline state. However, the reflectance was not provided, and the quality factor is very low (∼5) due to the inhomogeneous refractive index environment.

In this work, we propose a novel nonvolatile and reconfigurable mid-infrared bandpass filter based on tunable SLR supported by a two-dimensional (2D) array of gold nanorods, which are embedded in a thick Ge${}_{2}$Sb${}_{2}$Te${}_{5}$ film. The operation principle will be elaborated. Results will show that the reflection spectra of the proposed structure can be dynamically tuned by changing the Ge${}_{2}$Sb${}_{2}$Te${}_{5}$ crystallization fraction, which can be achieved with a single nanosecond laser pulse [8]. Remarkably, we will show that our design has extremely large spectral tunability of 1.598 μm (from 3.197 μm to 4.795 μm), which covers the majority of the mid-infrared regime, high reflection efficiencies and relatively large quality factors (R=72.6% and Q=19.6 at 3.197 μm, R=25.8% and Q=10.6 at 4.795 μm). The underlying physics will be clarified with near-field distributions as well as the strong dependence of the reflection spectra on the lattice period and the incidence angle. By investigating the effects of gold nanorod sizes, we will also show that the filtering performance can be further adjusted to achieve an even larger quality factor or peak reflectance.

## 2. Theory and Simulation Setup

**Figure 1:**
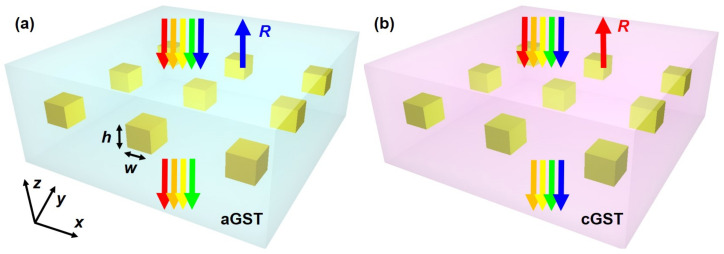
Schematic of the proposed tunable mid-infrared bandpass filter composed of Au nanorod array embedded in a thick Ge${}_{2}$Sb${}_{2}$Te${}_{5}$ film. When Ge${}_{2}$Sb${}_{2}$Te${}_{5}$ transits from (**a**) the amorphous phase (denoted as “aGST”) to (**b**) the crystalline phase (denoted as “cGST”), the narrowband reflection spectrum experiences a large red shift.

Figure 1 illustrates the proposed nonvolatile reconfigurable narrowband mid-infrared filter. The design is composed of a 2D array of gold nanorods that are embedded in an optically thick Ge${}_{2}$Sb${}_{2}$Te${}_{5}$ film. Here we adopted gold since it has a low loss in the mid-infrared regime and the advantage of being chemically stable in many environments [28]. The square-shaped gold nanorods have a side length of w=200 nm and thickness of h=180 nm, and the lattice periods along both the *x* and *y* axes are equal to Λ. The structure is illuminated by a normally incident plane wave with unitary electric field amplitude and linear polarization along the *x* direction.

The operation principle of the proposed filter is as follows. For such a gold lattice surrounded by homogeneous Ge${}_{2}$Sb${}_{2}$Te${}_{5}$ environment, the Rayleigh anomaly wavelength of the (p,q) diffraction order, $\lambda_{\mathrm{RA}}^{\left(p,q\right)}$, can be calculated by

(1) $$\begin{array}{c} k_{0}n=\left|{\vec{k}}_{\|}+\vec{G}\right|\;, \end{array}$$

where ${\vec{k}}_{\|}=k_{0}n\sin\theta$ with $k_{0}=2\pi /\lambda_{\mathrm{RA}}^{\left(p,q\right)}$ and θ the incidence angle, *n* is the refractive index of Ge${}_{2}$Sb${}_{2}$Te${}_{5}$, and $\vec{G}=\left(p\frac{2\pi }{\Lambda },q\frac{2\pi }{\Lambda }\right)$ is the reciprocal vector that is inversely proportional to the lattice period Λ. Thus under normal incidence (θ=0) we have

(2) $$\begin{array}{c} \lambda_{\mathrm{RA}}^{\left(p,q\right)}=n\Lambda /\sqrt{p^{2}+q^{2}}\;. \end{array}$$

**Figure 2:**
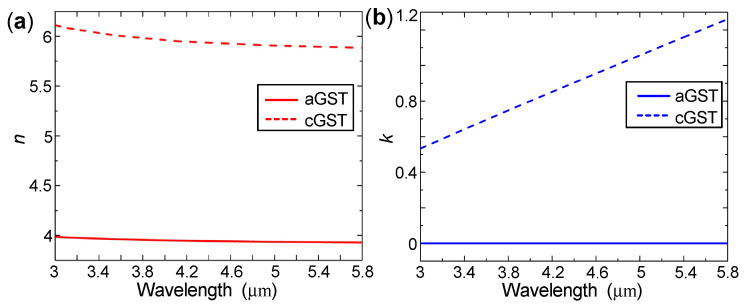
(**a**) Real and (**b**) imaginary parts of Ge${}_{2}$Sb${}_{2}$Te${}_{5}$’s refractive index as a function of the wavelength [29]. Solid and dashed curves are for Ge${}_{2}$Sb${}_{2}$Te${}_{5}$ in the amorphous state (denoted as “aGST”) and in the crystalline state (denoted as “cGST”), respectively.

If the gold nanorod arrays are well-designed such that SLR can be excited at wavelength that is close to the RA wavelength of the (±1,0) order, $\lambda_{\mathrm{RA}}^{\left(\pm 1,0\right)}=n\Lambda$, the SLR wavelength can be tuned by ∼$(n_{c}-n_{a})\Lambda$ when Ge${}_{2}$Sb${}_{2}$Te${}_{5}$ transits from the amorphous state with refractive index $n_{a}$ to the crystalline state with refractive index $n_{c}$. In the mid-infrared regime, $n_{a}\approx 4$ and $n_{c}\approx 6$ [29], as shown by Figure 2, thus we can expect a large spectral tunability of ∼2Λ, which is twice of the lattice period Λ. By taking Λ=750 nm so that the RA wavelengths of the (±1,0) order are $n_{a}\Lambda \approx 3\;\mu$m and $n_{c}\Lambda \approx 4.5\;\mu$m for Ge${}_{2}$Sb${}_{2}$Te${}_{5}$ in the amorphous and the crystalline states, respectively, we are able to obtain extremely large spectral tunability reaching ∼1.5μm, which covers the vast majority of the mid-infrared 3 μm to 5 μm atmospheric window.

All the simulations were performed with a home-developed package for fully vectorial rigorous coupled-wave analysis (RCWA) following [30,31,32]. As a powerful tool for modelling periodic structures, the RCWA technique can calculate the reflectance and the transmittance, as well as the near-field electric and magnetic field distributions. The numbers of the 2D Fourier harmonics in our RCWA simulations were 31×31, which were confirmed to be enough to reach the convergence regime. The wavelength-dependent permittivities of gold were taken from [33]. The effective wavelength-dependent permittivity of Ge${}_{2}$Sb${}_{2}$Te${}_{5}$ in various crystallization conditions can be described by the Lorenz–Lorentz relation [34],

(3) $$\begin{array}{c} \frac{\varepsilon_{\mathrm{eff}}\left(\lambda \right)-1}{\varepsilon_{\mathrm{eff}}\left(\lambda \right)+2}=m\frac{\varepsilon_{c}\left(\lambda \right)-1}{\varepsilon_{c}\left(\lambda \right)+2}+\left(1-m\right)\frac{\varepsilon_{a}\left(\lambda \right)-1}{\varepsilon_{a}\left(\lambda \right)+2}\;, \end{array}$$

where *m* is the crystalline fraction of Ge${}_{2}$Sb${}_{2}$Te${}_{5}$, ranging from 0 to 1. $\varepsilon_{a}\left(\lambda \right)$ and $\varepsilon_{c}\left(\lambda \right)$ are the dielectric constants of Ge${}_{2}$Sb${}_{2}$Te${}_{5}$ in the amorphous (m=0) and in the crystalline (m=1) states, respectively, which are calculated through the wavelength-dependent refractive indices taken from [29], as shown by Figure 2.

## 3. Results and Discussion

### 3.1. Spectral Tunability

**Figure 3:**
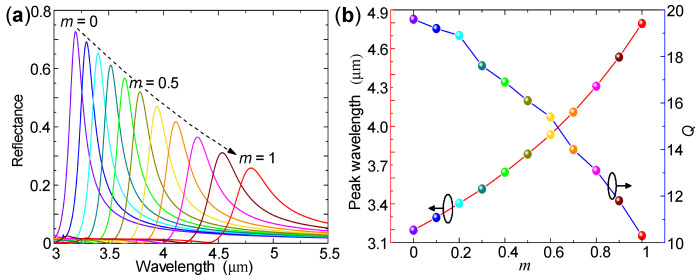
(**a**) Simulated reflection spectra of the proposed filter for different Ge${}_{2}$Sb${}_{2}$Te${}_{5}$ crystallization fractions ranging from 0 (the amorphous state) to 1 (the crystalline state) in step of 0.1. (**b**) Peak reflectance wavelength and quality factor as functions of Ge${}_{2}$Sb${}_{2}$Te${}_{5}$ crystallization fraction.

Figure 3a shows the calculated reflection spectra of the proposed filter for different Ge${}_{2}$Sb${}_{2}$Te${}_{5}$ crystalline fractions. Remarkably, results show that as the Ge${}_{2}$Sb${}_{2}$Te${}_{5}$ crystallization fraction *m* increases from 0 (the amorphous state) to 1 (the crystalline state), the reflection spectra are greatly red-shifted: the wavelength for the peak reflectance shifts from 3.197 μm to 4.795 μm. This corresponds to extremely large spectral tunability of 1.598 μm, or 2.13Λ, which is slightly larger than the RA wavelength tunability of ∼2Λ. This striking spectral tunability is larger or even much larger than most of the reported Ge${}_{x}$Sb${}_{y}$Te${}_{z}$-based mid-infrared filters, including those based on perfect absorbers [13,14,15,16], Fabry-Pérot cavities [18,19], extraordinary optical transmission effects [8,20], or SLRs combined with thin Ge${}_{x}$Sb${}_{y}$Te${}_{z}$ films [26,27].

On the other hand, as the Ge${}_{2}$Sb${}_{2}$Te${}_{5}$ crystalline fraction increases from m=0 to 1, the peak reflectance decreases from 72.6% to 25.8%, the linewidth increases from 163 nm to 467 nm, and the corresponding quality factor decreases from 19.6 to 10.3, as shown by Figure 3. The obtained quality factors are larger than those based on perfect absorbers [13,14,15,16], and are comparable or larger than those based on extraordinary optical transmission effects [20,21].

As summarized in Table 1, compared with the literature, our design has extremely large spectral tunability, which covers the majority of the mid-infrared regime (3 μm to 5 μm). It also has a relatively high reflection efficiency and meanwhile a relatively large quality factor.

### 3.2. Physics Mechanisms

**Figure 4:**
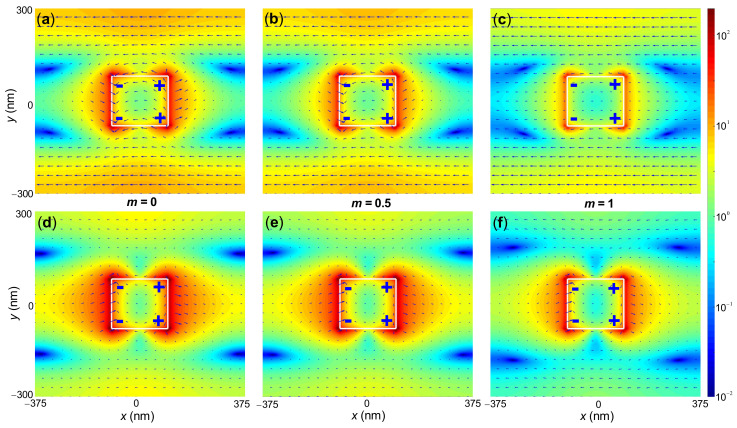
Simulated steady-state electric field intensity (in color) and vector (in arrows) maps at the three wavelengths of (**a**,**d**) 3.197 μm, (**b**,**e**) 3.785 μm, and (**c**,**f**) 4.795 μm, corresponding to peak reflectance for m=0, 0.5 and 1, respectively. (**a**–**c**) Top view at the central plane of gold nanorod (z=0), and (**d**–**f**) side view at y=0. The square-shaped gold nanorods surrounded by Ge${}_{2}$Sb${}_{2}$Te${}_{5}$ are outlined by white boxes. “+” and “−” indicate charge distributions.

In order to understand the physics underlying the large spectral tunability and the high quality factors of the designed filter, in Figure 4 we plot the simulated steady-state near-field electric field distributions at three wavelengths of 3.197 μm, 3.785 μm and 4.795 μm, which correspond to the peak reflectance for m=0, 0.5 and 1, respectively. For all these three wavelengths, results show that in-plane dipoles are excited in the gold nanorod and that the electric fields are greatly enhanced over a large volume, suggesting the excitation of in-plane SLRs. Moreover, the electric field distributions show particular curvatures inside and outside the gold nanorod and are strongly asymmetric in the *z* direction, consistent with the literature of SLRs [24,35,36].

As the Ge${}_{2}$Sb${}_{2}$Te${}_{5}$ crystallization fraction *m* increases from 0 to 1, the electric field intensities for the SLR wavelengths decrease, indicating weaker inter-nanorod interactions. Indeed, because the imaginary part of the Ge${}_{2}$Sb${}_{2}$Te${}_{5}$ refractive index increases dramatically as *m* increases from 0 to 1, as shown by Figure 2b, the collective coupling (via diffraction) between localized surface plasmon resonances that are supported by gold nanorods suffers from larger absorption loss in Ge${}_{2}$Sb${}_{2}$Te${}_{5}$. Therefore, as *m* increases, the SLR becomes weaker, corresponding to smaller peak reflectivity.

**Figure 5:**
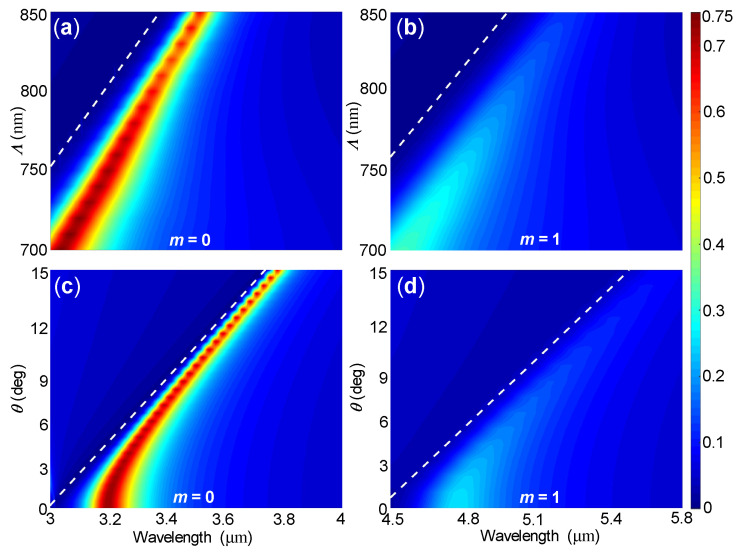
Simulated reflection spectra of the proposed filter with Ge${}_{2}$Sb${}_{2}$Te${}_{5}$ in (**a**,**c**) the amorphous state or (**b**,**d**) the crystalline state as functions of (**a**,**b**) the lattice period under normal incidence (θ=0) and (**c**,**d**) the incidence angle for Λ=750 nm. The white dashed curves represent RA wavelengths of the (±1,0) order, which were calculated with Equation (1).

In order to further validate the excitation of SLRs, we also calculated the reflection spectra of the proposed filter with Ge${}_{2}$Sb${}_{2}$Te${}_{5}$ in the amorphous state (m=0) or in the crystalline state (m=1) as functions of the lattice period Λ and the incidence angle θ. As Λ increases from 700 nm to 850 nm, Figure 5a,b show that the peak reflectance decreases for both m=0 and m=1, and that the corresponding wavelengths increase linearly from 3.035 μm to 3.515 μm for m=0, and from 4.555 μm to 5.275 μm for m=1. Therefore, the spectral tuning ranges are 1.52 μm and 1.76 μm, corresponding to 2.17Λ and 2.07Λ, respectively. Similarly, as θ increases from $0^{\circ }$ to $15^{\circ }$, Figure 5c,d also show that the peak reflectance decreases and that the corresponding wavelength increases for both m=0 and m=1. We note that the peak reflectance wavelengths generally follow the RA wavelengths of the (±1,0) order, confirming the excitation of SLRs. Additionally, the linewidths under oblique incidences are narrower than that under normal incidence, suggesting that the SLRs excited under oblique incidence have larger quality factors. This is consistent with the fact that out-of-plane SLRs excited under oblique incidences have larger quality factors than in-plane SLRs excited under normal incidence [37].

### 3.3. Effects of Gold Nanorod Sizes

**Figure 6:**
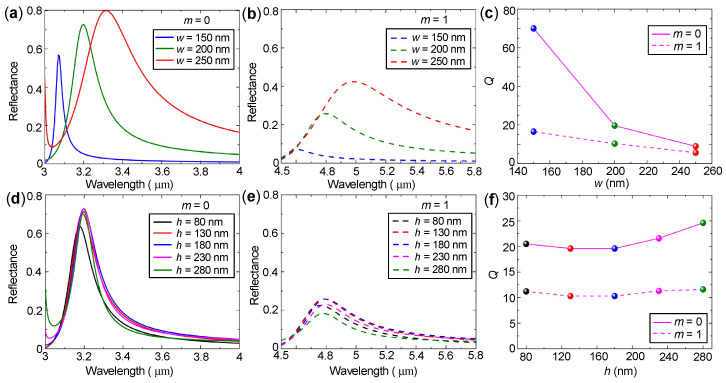
(**a**,**b**) Simulated reflection spectra and (**c**) quality factors of the proposed filter as functions of side length of gold nanorods. (**d**,**e**) Simulated reflection spectra and (**f**) quality factors of the proposed filter as functions of thickness of gold nanorods. Ge${}_{2}$Sb${}_{2}$Te${}_{5}$ in (**a**,**d**) is of amorphous state (m=0) and in (**b**,**e**) is of crystalline state (m=1). The calculations were performed with Λ=750 nm, h=180 nm for (**a**–**c**), and w=200 nm for (**d**–**f**).

We now study the effects of the gold nanorod sizes on the filtering performance. Figure 6a,b shows that if the side length of gold nanorods *w* decreases from 200 nm (blue curves) to 150 nm (purple curves), the peak reflectance decreases slightly from 72.6% to 56.8% for Ge${}_{2}$Sb${}_{2}$Te${}_{5}$ in the amorphous state (m=0), but decreases dramatically from 25.8% to 7.1% for Ge${}_{2}$Sb${}_{2}$Te${}_{5}$ in the crystalline state (m=1). At the cost of decreased reflectance, the corresponding quality factors increase tremendously from 19.6 to 70 for m=0, and from 10.3 to 16.5 for m=1, as shown in Figure 6c. If *w* increases to 250 nm (red curves), the peak reflectance further increases from 72.6% to 80.0% (or from 25.8% to 42.5%) at the cost that the quality factor decreases from 19.6 to 8.9 (or from 10.3 to 5.6) for m=0 (or m=1). Therefore, by carefully designing the gold nanorod side length, the peak reflectance can be greatly increased at the cost of the relatively smaller quality factor or the quality factor can be significantly improved at the cost of relatively smaller peak reflectance. This adjustable filtering performance makes our design very attractive because it can meet various demands.

If the thickness of gold nanorods *h* increases or decreases by the same amount of 50 nm or even by 100 nm, however, Figure 6d–f show that the peak reflectance, as well as the quality factor, have negligible variations for both m=0 and m=1. In other words, *h* has little influence on the filtering performance. Such extremely large tolerance on the gold nanorod thickness will greatly facilitate the fabrication of the proposed filter.

## 4. Conclusions

In conclusion, we have proposed a nonvolatile, reconfigurable, and narrowband mid-infrared bandpass filter based on SLRs in phase-change material Ge${}_{2}$Sb${}_{2}$Te${}_{5}$. Results have shown that when Ge${}_{2}$Sb${}_{2}$Te${}_{5}$ transits from the amorphous state (m=0) to the crystalline state (m=1), the narrowband reflection spectrum of the proposed filter can be tuned from 3.197μm to 4.795μm, covering the vast majority of the mid-infrared 3 μm to 5 μm atmospheric window. For m=0 the peak reflectance reaches 72.6% and the quality factor is up to 19.3, while for m=1 the peak reflectance reaches 25.8% and the quality factor is 10.3. Near-field distributions, and the strong dependence of the reflection spectra on the lattice period and the incidence angle have confirmed that the narrowband filtering originates from the excitation of SLR. We have also found that the quality factor can be greatly increased to Q=70 for m=0 (or Q=16.5 for m=1) at the cost of relatively smaller peak reflectance, and the peak reflectance can be improved to 80.0% for m=0 (or 42.5% for m=1) at the cost of relatively smaller quality factors. We expect that the designed nonvolatile reconfigurable narrowband mid-infrared filters will find applications in chemical spectroscopy, thermography, and multispectral/hyperspectral imaging. We also expect that dynamically tunable SLRs based on phase change materials can be extended to other spectral regimes and to other applications besides filtering.

## Figures and Tables

**Table 1:** Performance comparison on spectral tunability, efficiency and quality factor between the literature and this work.

Work	Spectral Tunability	Efficiencies	Quality Factors
Ref. [8]	2.91 μm–3.41 μm	70–70%	45–45
Ref. [13]	2.23 μm–2.46 μm	96–80%	44.6–61.5
Ref. [19]	4.26 μm–4.55 μm	60–75%	70–90
Ref. [26]	1.89 μm–2.27 μm	17–28%	17–13
This work	3.179 μm–4.795 μm	72.6–25.8%	19.6–10.3
